# The Next Frontier of Regulatory T Cells: Promising Immunotherapy for Autoimmune Diseases and Organ Transplantations

**DOI:** 10.3389/fimmu.2020.565518

**Published:** 2020-09-23

**Authors:** Lauren V. Terry, Ye Htun Oo

**Affiliations:** ^1^Centre for Liver and Gastrointestinal Research, National Institute for Health Research Birmingham Biomedical Research Council, Institute of Immunology and Immunotherapy, University of Birmingham, Birmingham, United Kingdom; ^2^European Reference Network (ERN) Centre—Rare Liver, Queen Elizabeth Hospital, Birmingham, United Kingdom; ^3^Liver Transplant Unit, University Hospital of Birmingham National Health Service Foundation Trust, Birmingham, United Kingdom

**Keywords:** regulatory T cell, liver transplant, autoimmune liver diseases, antigen specific, recruitment, tolerance, polyclonal

## Abstract

Regulatory T cells (Tregs) are crucial in maintaining tolerance. Hence, Treg immunotherapy is an attractive therapeutic option in autoimmune diseases and organ transplantations. Currently, autoimmune diseases do not have a curative treatment and transplant recipients require life-long immunosuppression to prevent graft rejection. There has been significant progress in understanding polyclonal and antigen-specific Treg biology over the last decade. Clinical trials with good manufacturing practice (GMP) Treg cells have demonstrated safety and early efficacy of Treg therapy. GMP Treg cells can also be tracked following infusion. In order to improve efficacy of Tregs immunotherapy, it is necessary that Tregs migrate, survive and function at the specific target tissue. Application of antigen specific Tregs and maintaining cells' suppressive function and survival with low dose interleukin-2 (IL-2) will enhance the efficacy and longevity of infused GMP-grade Tregs. Notably, stability of Tregs in the local tissue can be manipulated by understanding the microenvironment. With the recent advances in GMP-grade Tregs isolation and antigen-specific chimeric antigen receptor (CAR)-Tregs development will allow functionally superior cells to migrate to the target organ. Thus, Tregs immunotherapy may be a promising option for patients with autoimmune diseases and organ transplantations in near future.

## Background of Regulatory T Cells

Naturally occurring CD4^+^CD25^high^ regulatory T (Treg) cells maintain peripheral self-tolerance in rodents and humans (1, 2). In 1995, Sakaguchi and colleagues demonstrated that adoptive transfer of a subset of CD4^+^ T cells expressing the IL-2 receptor α-chain (CD25) prevented autoimmunity (1). CD4^+^CD25^high^ T cells constitute 5 to 10% of CD4^+^ T cells in the blood (3) and they are able to maintain immunologic self-tolerance and transplantation tolerance by actively suppressing self-reactive, alloantigen-reactive lymphocytes. Treg cells prevent activation and expansion of auto-reactive T cells that escape clonal deletion in the thymus.

Treg cell development and function is controlled by the transcription factor Foxp3, however defects can lead to autoimmune and inflammatory syndromes in humans and mice (4). Immunodysregulation, polyendocrinopathy, X-linked (IPEX) syndrome, a rare X-linked recessive disorder, was first described in 1982 in which mutations in the Foxp3 gene caused defective development of CD4^+^CD25^high^ Treg cells (5). Similarly, lymphoproliferation and multi-organ autoimmunity in scurfy mutant mice was caused by mutations in Foxp3 (6). Further to this, expression of the IL-7 receptor, CD127, correlated inversely with Foxp3 expression and Treg cell suppressive function; hence Treg cells are currently defined as CD4^+^CD25^high^CD127^low^Foxp3^+^ cells (7, 8).

Treg cells main function is to control auto-reactive T cells via multiple mechanisms. Treg cells express CTLA-4 and suppress effector T cells, via endocytosis of CD80/CD86 molecules expressed on antigen presenting dendritic cells (9). They can also secrete immunosuppressive cytokines (interleukin-10), cytotoxic granzymes and immunomodulatory molecule, kynurenine. Treg cells can generate the immunosuppressive molecule, adenosine, via CD39 expressed on the cell surface (8). By continuously surveying and controlling self-reactive T effector cells, Treg cells are crucial in restoring tolerance in both autoimmunity and transplantation.

## Types of Regulatory T Cells

CD4^+^CD25^high^ Foxp3^+^ Treg cells are a heterogeneous population, often categorized into two main subtypes, which include thymic-derived natural Treg (tTreg) cells and peripheral-derived Treg (pTreg) cells (10, 11). Both subtypes act in a complementary manner to maintain peripheral tolerance. tTreg cells express Foxp3, however Foxp3 is transiently induced in pTreg cells; they may not have high levels of Treg specific demethylated region (TSDR), a conserved region within the Foxp3 gene (12). pTreg cells recognize non-self-antigens, such as those encountered in the gut and airways, in addition to providing maternal-fotal tolerance and commensal micro-biota tolerance (13–15). pTreg cells develop from conventional T cells once they have been exposed to non-self-antigens and transforming growth factor-β (16, 17). Although tTreg cells and pTreg cells can be distinguished by using epigenetics markers, phenotypically they share the same surface markers, which poses difficulty in specifically isolating tTreg cells for subsequent therapeutic purposes.

## Interleukin-2

Treg cell survival and function is dependent on the cytokine, interleukin-2 (IL-2) (18), which is required for maintaining effective levels of functional Treg cells in autoimmune disease (AID) (19–21). The cell surface receptor for IL-2 (IL-2R) is composed of three subunits, alpha (IL-2RA, CD25), beta (IL-2RB, CD122), and gamma (IL-2RG, CD132). Treg cells constitutively express IL-2RA. IL-2RA is required for high-affinity IL-2 binding, whilst IL-2RB and IL-2RG transduce the IL-2 signal (22). Owing to their high levels of high-affinity CD25, Treg cells competitively consume IL-2, thereby maintaining their survival and function, whilst suppressing bystander effector cells (23, 24). Where IL-2 availability is low, such as in the inflamed hepatic microenvironment, Treg cell function may be compromised and be inadequate to counteract the activated immune infiltrate (25). At tissue level, Treg cell suppression is via IL-2 and CTLA-4 dependent mechanisms, yet tissue Treg cells, such as intrahepatic Treg cells, constitutively express CTLA-4 (25, 26). CTLA-4 can capture its ligands, CD80 and CD86, from antigen presenting cells (APCs) by trans-endocytosis, there by acting as an effector molecule to inhibit CD28 co-stimulation by the cell-extrinsic depletion of ligands (9).

## Autoimmune Diseases

**Figure 1:**
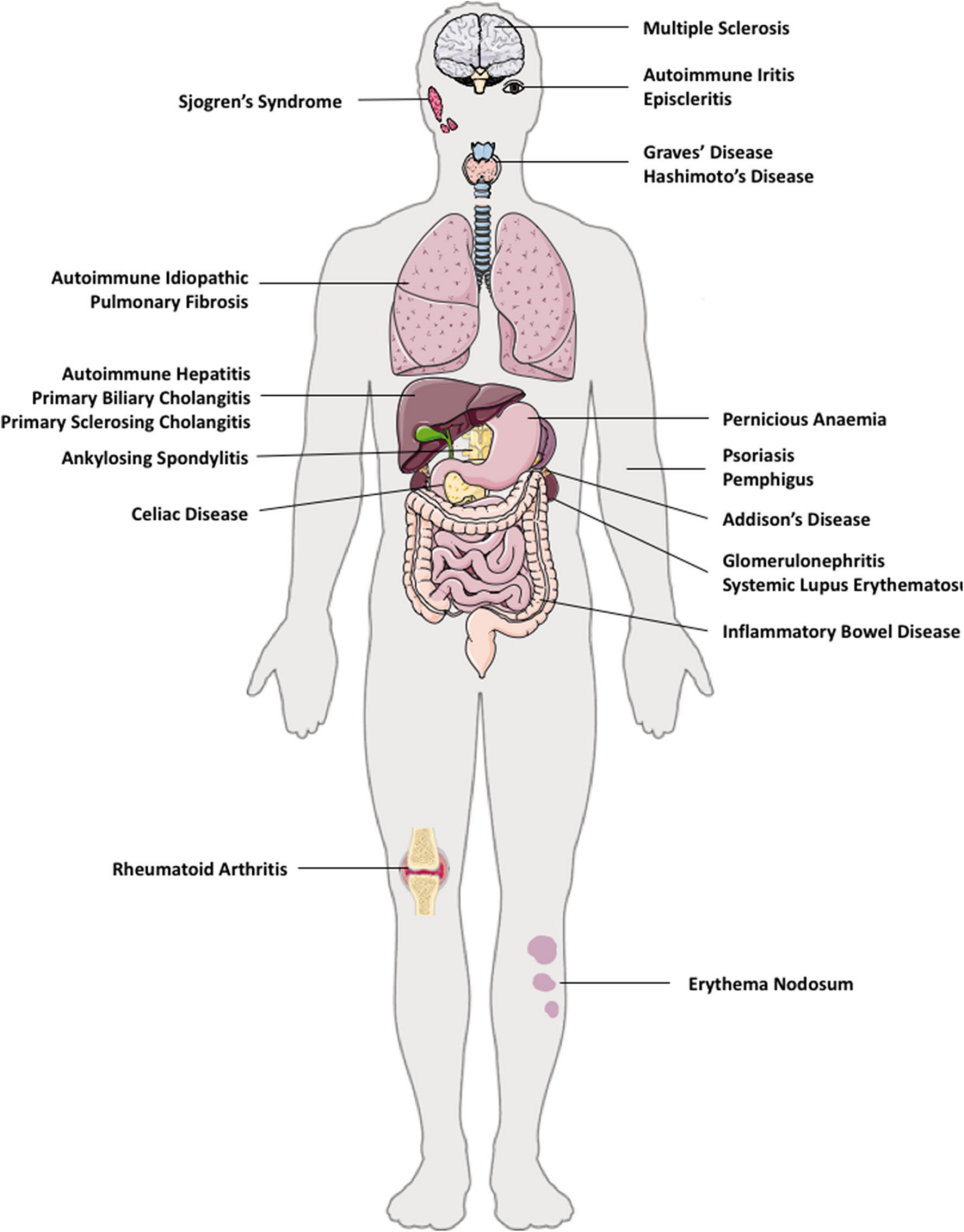
Autoimmune diseases in multiple organs in humans. e.g. autoimmune diseases that can co-exist in multiple tissues are: brain (multiple sclerosis), eye (autoimmune iritis and episcleritis), lung (autoimmune idiopathic pulmonary fibrosis), gut (Coeliac disease, pernicious anemia, inflammatory bowel disease), liver—autoimmune liver diseases (autoimmune hepatitis, primary biliary cholangitis, primary sclerosing cholangitis), kidneys (autoimmune glomerulonephritis), skin (psoriasis, pemphigus), endocrine (autoimmune thyroid disease such as Hashimoto thyroiditis, Graves' disease, type 1 diabetes, Addison's disease), and multi-organ involvement, such as systemic lupus erythematosus.

The pathogenesis of AID is attributed by genetic susceptibilities, environmental factors, and gut micro-biota (27). The majority of organs and tissues in humans are susceptible to AIDs; such as in solid organs (liver–autoimmune liver diseases and kidneys–autoimmune glomerulonephritis), brain (multiple sclerosis), lung (autoimmune idiopathic lung fibrosis), gut (Coeliac disease, pernicious anemia, inflammatory bowel diseases), skin (psoriasis, pemphigus, erythema nodosum), endocrine (autoimmune thyroiditis, diabetes, Addison's disease), and multi-organ involvement, for example, systemic lupus erythematosus (SLE) (Figure 1).

Autoimmune liver diseases (AILDs) include autoimmune hepatitis (AIH), primary biliary cholangitis (PBC) and primary sclerosing cholangitis (PSC) (28, 29). Both AIDs and AILDs are immune mediated diseases of unknown etiology, and occur as a result of a breakdown in immune homeostasis (30). Immunological homeostasis in humans is maintained between a balance of effector T cells and Treg cells. It is now accepted that there is a lack of control of self-reactive T effectors cells by Treg cells in AIDs (31). Most AIDs co-exist with other autoimmune conditions in the same individual (Figure 1), therefore, restoring the tolerance in one autoimmune condition may also alleviate other AIDs, if the origin of initial pathology stems from the same tissue.

Therapies targeting AIDs, often aim to inhibit pro-inflammatory immune responses by preventing activation of immune cells in the target organ or diminishing tissue specific T cell populations (32). Treg cells play a role in restoring multiple human liver autoimmune conditions, however, Treg-directed therapies could potentially inhibit protective immune responses and lead to a higher risk of susceptibility to infection (31). As Treg cells have significant immune-homeostatic properties to prevent autoimmunity in mice and human, Treg-based cellular therapies have gained extensive interest in the past decade. Yet, most approaches to suppress autoimmunity currently use polyclonal Treg cells due to their success in animal models.

Polyclonal Treg cells have several limitations, such as the possibility of inducing global immunosuppression and predisposing patients to infection. These limitations may be overcome by using auto-antigen specific Treg cells. Most investigators are currently focusing on the suppression of auto-reactive T cells in an antigen-specific manner (33, 34). The use of T cell receptor (TCR) transgenic mice confirmed that antigen-specific Treg cells are more potent and efficacious in autoimmune diabetes (35). Although antigen-specific Treg cells provide a new direction for Treg cellular therapy, the challenges remain in isolating and expanding antigen-specific Treg cells, in addition to a lack of understanding of their mechanism of suppression, survival and plasticity. These aspects require addressing before embarking on organ-specific Treg cellular therapy.

## Polyclonal Regulatory T Cells and Low Dose IL-2 Therapy in Autoimmune Disease

In healthy individuals, antigens can be efficiently eliminated without damaging their own tissue, due to the removal or inactivation of self-reactive cells, without posing a threat to the individual (36). Yet, when there is a breakdown in peripheral tolerance, the control of self-reactive cells can become dysregulated and aberrant immune responses can lead to harmful effects in their own tissue, which may lead to the development of AID (8). Reduction in the frequency of Treg cells or a decline in their function has been reported in various autoimmune condition settings (37). Failure of Treg cells to suppress effector cells is a typical feature of autoimmunity and has led to studies exploring the use of either polyclonal (38, 39) or antigen-specific Treg cells (40–42) as cellular therapies for AIDs (43).

AIDs can affect specific organs of the body (Figure 1). Multiple sclerosis is a debilitating and known AID of the nervous system. As mentioned, AILD includes AIH, PBC, and PSC. Autoimmune endocrine diseases include autoimmune thyroiditis (both Hashimoto and Grave's disease) Sjogren's syndrome, and type-1 diabetes mellitus. Autoimmune musculo-skeletal diseases are ankylosing spondylitis, rheumatoid arthritis, pemphigus and psoriasis. Further to this, gastrointestinal autoimmune conditions comprise of coeliac disease, pernicious anemia, and inflammatory bowel diseases (Figure 1). AIDs can also affect multiple organs and tissues in the same patient, for example, autoimmune PBC patients often have associated Sjogren's syndrome and autoimmune thyroid disease (28).

Adoptive Treg cell therapy has been applied in SLE patients with active skin disease (44). The study demonstrated that Treg cell accumulation in the skin was associated with a marked attenuation of the interferon-γ pathway and a reciprocal augmentation of the interleukin-17 pathway (44). Additionally, good manufacturing practice (GMP) Treg cell therapy in children with another autoimmune condition, *Type 1 diabetes mellitus*, demonstrated that Treg therapy was safe without any serious side effects, at a Treg cell dose ranging from 10–20 millions/Kg. Children were followed up for a year and the trial revealed that there was a lower insulin requirement and higher C peptides compared to the control patient group (39, 45). A recent trial from UCSF on adult patients with diabetes that utilized GMP polyclonal Treg cells ranging from 5 million to 2.6 billion showed that Treg cell therapy is safe and that there was stable C peptide levels and insulin for 2 years after GMP Treg cell therapy. Cells were labeled with Deuterium and could be traced in the circulation for up to 12 months (38). In addition, CD4^+^CD25^+^CD127^low^CD45RA^+^ Treg cells have been suggested as the most suitable subset for GMP Treg cell trial in *inflammatory bowel disease* (46). Thus, there are many investigators attempting to restore tolerance in AIDs with direct application of Treg cells to patients.

In addition to GMP-Treg cell infusion, Treg cell enhancing therapy with very low dose IL-2, has also been applied in various AIDs. In *HCV induced vasculitis* (47), this therapy has been shown to be safe and led to Treg cell recovery with concomitant clinical improvements in patients who had cutaneous vasculitis. Additionally, a randomized double blind clinical trial using subcutaneous low dose IL-2 on alternate days for 2 weeks, followed by a 2-week break at a dose of 1 million IU or placebo, in patients with active SLE, suggested that low dose IL-2 was again safe and may be an effective therapy (48). Immunological and clinical efficacy of low dose IL-2 was assessed in a recent open-label, phase I-IIa study of 46 patients with *multiple autoimmune conditions*: including rheumatoid arthritis, ankylosing spondylitis, SLE, psoriasis, Behcet's disease, granulomatosis with polyangiitis, Takayasu's disease, Crohn's disease, ulcerative colitis, AIH or PSC. All patients received low dose IL-2 (1 million IU/day) for 5 days, followed by fortnightly injections for 6 months. The results suggested that low dose IL-2 was well tolerated in all AIDs, in addition to observing Treg cell expansion and activation in all patients, without effector T cell activation (49, 50).

## Polyclonal Regulatory T Cells and Low Dose IL-2 Therapy in Autoimmune Liver Disease

As previously mentioned, AILDs include AIH, which affects hepatocytes, PBC and PSC, which affects bile ducts. Functional impairment or quantitative deficiency of Treg cells has been described in AILD (25, 51, 52). In AIH, hepatocytes, which constitute around 70% of liver cells, are mainly damaged by auto-reactive T cells (51, 53–55). PBC affects the intrahepatic bile ducts and PSC affects both the intra- and extrahepatic bile ducts and is associated with inflammatory bowel disease. Current therapies for AILD are non-curative, provide unsatisfactory control of hepatic and biliary inflammation and require long-term immunosuppressive medications that carry unfavorable side effects. Thus, autologous Treg cell therapy is an attractive option for the treatment of AILD, which could provide long-term immune-regulation without requiring global immunosuppression.

To work effectively, adoptively transferred Treg cells must migrate to and mediate suppression of auto-reactive T cells at the targeted tissue. Chemokines direct the trafficking and positioning of leukocytes within the tissue by attracting T cells, which express the corresponding chemokine receptors (56, 57). Deficiency of chemokine receptor, CXCR3, which drives recruitment of T cells across hepatic sinusoids (25, 58) has been associated with the exacerbation of liver disease and abrogation of tolerance in mouse models of immune-mediated hepatitis (59). A recent report by Oo and colleagues described that around 22–44% of infused GMP Treg cells migrate to the human autoimmune liver via utilizing CXCR3 (60). There is also another registered Phase 1 clinical trial to treat AIH patients with Treg cells (NCT02704338) yet the result is still awaited.

The stability of Treg cells at the site of hepatic inflammation is crucial. Our group has previously demonstrated that Treg cells expressing Treg Th1 phenotype are more common in an inflamed liver microenvironment (25). In addition, our recent data suggested that the human liver has low levels of IL-2 that may not support Treg cell survival (26). Based on this data, low dose IL-2 was administered in two patients with refractory AIH and an increase in the frequency of Treg cells in the peripheral circulation was observed (61).

## Organ Transplantations

**Figure 2:**
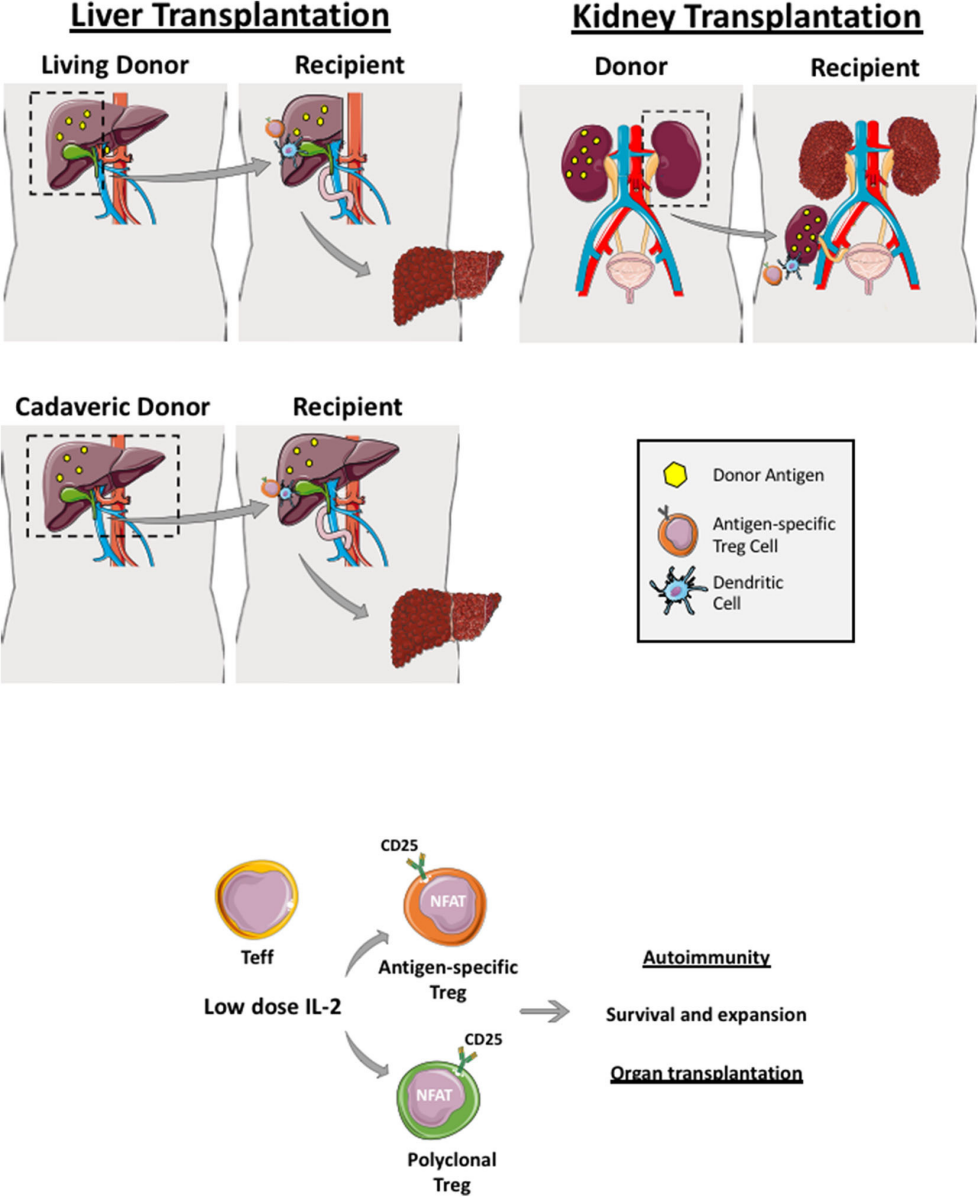
Regulatory T cells in live and cadaveric liver transplant. Liver transplantation can be a living-related (right lobe graft- upper figure) or a cadaveric transplant (whole liver transplant—lower figure). Renal transplantation can also be cadaveric or a live donor allograft. If it is a living donor transplantation, antigen-specific Treg cells can be generated and expanded before transplantation whereby recipient Treg cells are co-cultured with donor dendritic cells, which are primed with donor antigens (yellow dots). Both polyclonal and antigen-specific Treg cells proliferate and function via utilizing interleukin-2 as Treg highly express IL-2 receptor, CD25. These generated Treg cells are applied in both autoimmune disease and transplantation clinical trials.

**Figure 3:**
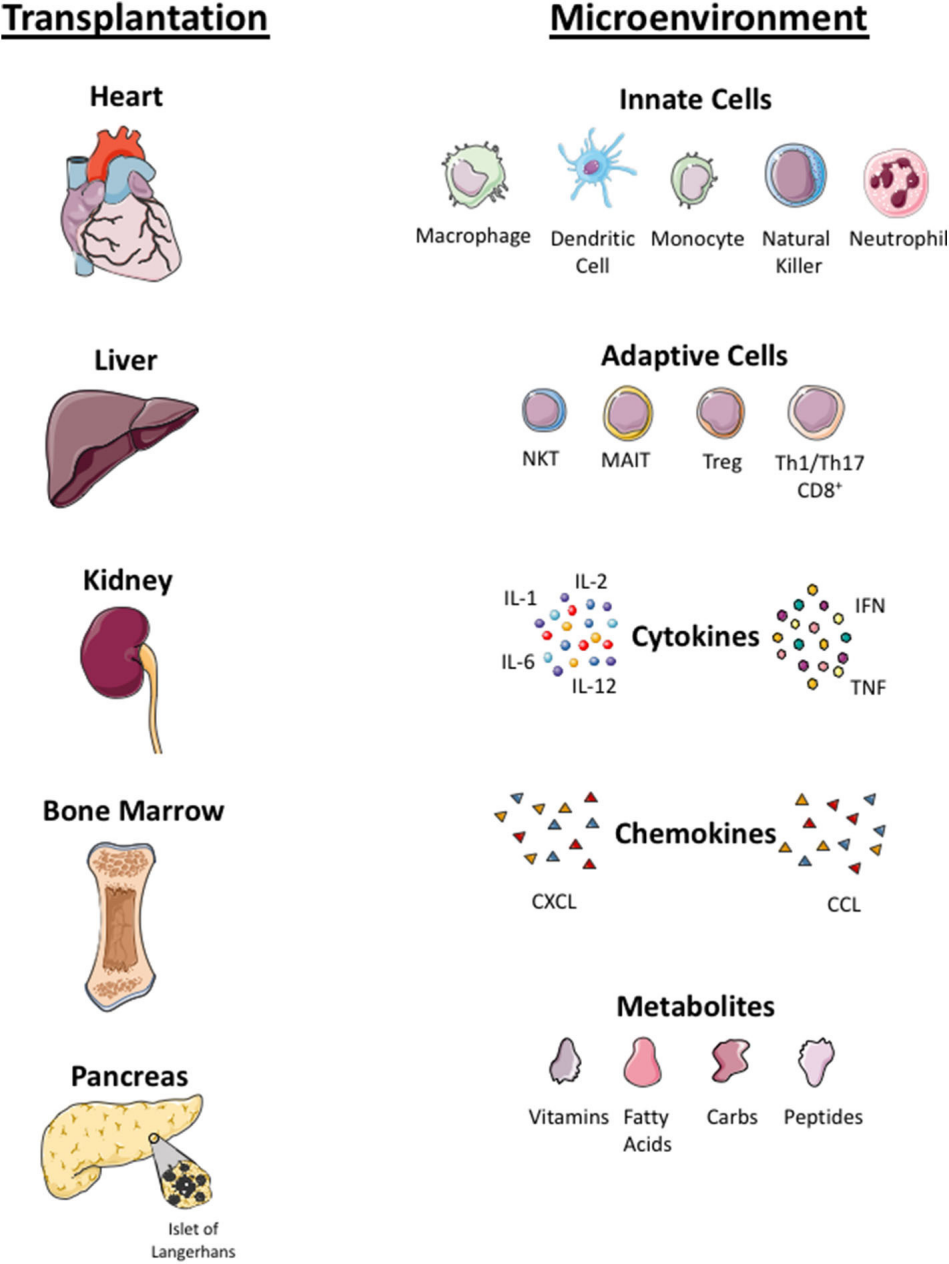
Microenvironmental factors in different organs. Local tissue microenvironmental factors in organ (heart, liver, kidney, bone marrow, islet cells) transplantation. Infused Treg cells arrive to local tissue where they crosstalk with innate immune cells (macrophage, dendritic cells, monocyte, natural killer cells, neutrophils) and adaptive immune cells (NKT, Mucosa associated invariant T cells, Treg, Th1, Th17 cells) in the local tissue. Treg cells localize in tissue with pro-inflammatory cytokines (interleukin 1, 6 and 12, tumor necrosis factor (TNF) and interferon (IFN)), chemokines (CXCL and CCL) and local metabolites (carbohydrate, fatty acid, peptides and vitamins). Infused Treg cell plasticity and their function in the local tissue microenvironment is dependent on these factors.

Transplantation provides the best chance of survival in organ failure or cancer and has become a routine clinical treatment option for many patients. Bone marrow, heart, liver, and kidney transplantations are performed in many countries however, recently the transplantation field has progressed to islet cell and multi-visceral transplantations in some centers (Figures 2, 3). Due to the current demand of organ donors exceeding the number of transplantable organs, live donation (liver and kidneys), and split graft transplants (liver) have become a vital option (62).

In the early period following transplantation, both direct and indirect pathways of antigen presentation by donor and recipient dendritic cells play a role in graft rejection (63). Transplant recipients are required to take long-term immunosuppression to prevent graft loss. Graft rejection is driven by an imbalance in the adaptive T and B immune cells: mainly a decline in the frequency of Treg cells or impairment in Treg cell function, which controls alloantigen specific T cells. To prevent graft rejection in both live and cadaveric transplantation, switching the immune balance toward the regulatory arm with Treg cellular therapy has become an exciting therapeutic option. Two main aims of Treg immunotherapy are 1) to restore immune tolerance and 2) to avoid life-long immunosuppression following transplantation. The side effects of immunosuppression range from sepsis, hypertension, diabetes, and renal dysfunction, to, the long term-effects of malignancies and post-transplant lymphoproliferative disorder (PTLD).

In the context of living-related liver and kidney transplantation, Treg cell infusion could be planned in advance. For example, recipient Treg cells could be isolated several weeks prior to the organ transplant and expanded with donor-derived APCs (dendritic cells, monocytes or B cells). This would allow sufficient numbers to be obtained in order to infuse them back into the organ recipient within the first few days post-transplant when immunosuppression and microenvironment milieu is optimized (Figures 2 and 3). Recipient Treg generation could be 1) polyclonal (if recipient Treg cells are expended with CD3, CD28 beads, IL-2 and Rapamycin) or 2) antigen-specific (if recipient Treg cells are expanded with donor APCs primed with the donor antigen) (Figure 2). Thus, autologous, polyclonal or antigen-specific Treg cell therapy can be administered to the liver or kidney organ transplant recipient immediately following transplantation. This could potentially lead to prevention of early graft rejection with a potential withdrawal of life-long immunosuppression.

Multiple clinical trials are registered for polyclonal Treg cell immunotherapy or low dose IL-2 cytokine therapy, in autoimmune conditions and organ transplantations (clinicalTrials.gov) (Table 1). These trials are currently being carried out or completed with CD4^+^CD25^high^ or CD4^+^CD25^high^CD127^low^ populations, which are GMP cell-sorted and expanded with TCR stimulation, or subcutaneous administration of low dose IL-2 (Aldesleukin). So far, there is no clinical trial completed with antigen-specific Treg cell immunotherapy.

**Table 1:** Table summarizing clinical applications of Treg cells in liver and autoimmune diseases in current clinical trials.

**LIVER TRANSPLANTATIONS**						
	**Trial ID/** **Name**	**Lead institution**	**Expansion: donor-specific or polyclonal**	**Application dose**	**Mechanism of recovery**	**Status of trial**
Liver transplant (live donor) (2–6 years post-Tx)	NCT02474199 Donor Alloantigen Reactive Tregs (darTregs) for Calcineurin Inhibitor (CNI) Reduction (ARTEMIS)	University of California (UCSF), USA	Donor-specific	300–500 × 10^6^ cells intravenous infusion	Cell sorting of CD4^+^, CD25^++^, CD127^low^ Treg	Completed results awaiting
Liver transplant	NCT02188719 Donor-Alloantigen-Reactive Regulatory T Cell (darTregs) in Liver Transplantation (deLTa) dELTA	UCSF, USA	Donor-specific	4 cohorts dose escalation 25-960 × 10^6^ cells	Cell sorting of CD4^+^, CD25^++^, CD127^low^ Treg	Terminated
Liver transplant	NCT03654040 LITTMUS-UCSF	UCSF, USA	Donor-specific alloantigen-specific T regulatory cells (arTreg)	Target dose: 100-500 × 10^6^ cells	Cell sorting of CD4^+^, CD25^++^, CD127^low^ Treg	Not yet recruiting
Liver	NCT01624077 1st Trial	Nanjing, China	Polyclonal	1 × 10^6^/kg at intervals	Unknown	Unknown
Liver	NCT01624077 2nd Trial	Nanjing, China	Donor-specific (MHC peptides)	1 × 10^6^/kg at intervals	Unknown	Unknown
Liver	NCT02166177 ThRIL	King's College Hospital, UK	Polyclonal	0.5–6.5 × 10^6^/kg	CliniMACS CD4^+^ CD25^high^ Treg	Completed Safe, well tolerated
Liver Live-donor transplant	Todo Okumura	Hokkaido, Japan	Donor-specific and co-stimulation blockade	0.23–6.37 × 10^6^/kg CD4^+^ CD25^+^ Foxp3^+^ Treg cells		Completed Safe, immunosuppression withdrawal achieved in 7/10 patients
**AUTOIMMUNE DISEASES**						
Type 1 diabetes mellitus	NCT01210664	UCSF	Polyclonal Treg	5-2,600 × 10^6^ cells/kg		Safe, c peptide improved, insulin requirement decline, cell can be tracked for 12 months
Type 1 diabetes mellitus	NCT02772679	UCSF, USA	Polyclonal Tregs + IL-2 (TILT)	3–20 × 10^6^ cells and two 5-day courses of IL-2 (1 × 106 IU daily)	Cell sorting of CD4^+^, CD25^++^, CD127^low^ Treg	Active, not recruited yet
Type 1 diabetes mellitus	ISRCTN06128462	Medical University of Gdansk, Poland	Polyclonal	10–20 × 10^6^ cells/kg	Cell sorting of CD4^+^, CD25^++^, CD127^low^ Treg	Safe, well tolerated
Autoimmune hepatitis	AUTUMN	University of Birmingham, UK	Polyclonal	8.9–86 × 10^6^ cells	GMP CD4^+^ CD25^high^ CliniMACS isolation	Completed 22–44% of Treg home to autoimmune livers
Pemphigus Vulgaris	NCT03239470	UCSF	Polyclonal	1–2.5 × 10^8^ cells	Cell sorting of CD4^+^, CD25^++^, CD127^low^ Treg	Recruiting

## Polyclonal Regulatory T Cells and Low Dose IL-2 Therapy in Human Transplantations

Polyclonal Treg cells have been applied in bone marrow and solid organ transplantations with a view to either reduce immunosuppression or achieve transplant tolerance without requiring immunosuppression. Treg cells mediate suppression of anti-donor immune responses, thus Treg cell enrichment following bone marrow, liver, and kidney transplantation could potentially lead to immunosuppression-free operational tolerance.

Treg cells can be isolated and expanded in large scale from the peripheral blood and the umbilical cord blood, which have been infused back to patients and proved to be safe in graft vs. host disease (GvHD) clinical trials (64, 65). Additionally, Treg cells have been applied in renal transplantation clinical trials. In the context of bone marrow transplantation, adoptive Treg cell therapy has been applied to control GVHD by utilizing freshly isolated or *ex vivo* expanded CD4^+^CD25^+^ Treg cells (65–67).

The TRACT trial infused 500–5000 × 10^6^ CD4^+^ CD25^+^ Treg cells (>80% Foxp3^+^) to nine patients, 2 months after live donor renal transplantation (68). The immunosuppression regimens included Alemtuzumab, followed by mycophenolate mofetil (MMF) and Tacrolimus. During this trial, there were no reported cases of opportunistic infections or graft rejection. In addition, the UCSF team applied polyclonal Treg cells into renal allograft subclinical inflammation. This type of low-grade inflammation can lead to late graft rejection, thus it demonstrated the application of Treg cells in this context (69). The team recruited three patients with biopsy-proven inflammation and administered a dose of 320 million CD4^+^ CD25^+^ CD127^low^ Treg (>93% Foxp3^+^) cells. Patients were on MMF, tacrolimus, and prednisolone immunosuppression. Following infusion, deuterated glucose was applied into the culture media to track infused Treg cells in the circulation. There were no side effects noted after infusion, however deuterated cells were not detected at the site of renal inflammation.

More recently, the application of Treg cells in renal transplantation was studied in the ONE study (in press, Vol 395 May 23, 2020, Lancet). The ONE Study consisted of seven investigator-led, single-arm trials done internationally at eight hospitals in France, Germany, Italy, the UK, and the USA (60 week follow-up). Included patients were living-donor kidney transplant recipients aged 18 years and older. The reference group trial (RGT) was a standard-of-care group given basiliximab, tapered steroids, MMF, and tacrolimus. The trial has just been completed and investigators concluded that regulatory cell therapy is achievable and safe in living-donor kidney transplant recipients, and is associated with fewer infectious complications, but similar rejection rates in the first year. Therefore, immune cell therapy is a potentially useful therapeutic approach in recipients of kidney transplant to minimize the burden of general immunosuppression.

## Polyclonal Regulatory T Cells and Low Dose IL-2 Therapy in Liver Transplantation

Liver transplantation has now become a standard therapeutic option for patients with acute liver failure or end-stage chronic liver disease. The ideal situation would be to achieve operational tolerance, which is defined as successful withdrawal of immunosuppression and to maintain a stable, preserved graft function following transplantation (31, 70). However, less than 20% of liver transplant recipients will achieve operational tolerance (71). The majority of patients will require life-long immunosuppression in order to control recipient immune responses toward allogeneic major histocompatibility complex (MHC) antigens from the donor liver graft and to reduce the risk of graft rejection and graft loss (72). Whilst immunosuppression has drastically improved survival rates over the last five decades, there are detrimental side effects that impose substantial risks to transplant recipients, such as: opportunistic infections, increased susceptibility to sepsis, renal dysfunction, and an increased risk of *de novo* malignancy (73). Immunosuppressive medications generally used are steroids, MMF, Tacrolimus, and Sirolimus. These medications have different impacts on different immune cell subset, which has been described in detail, in Table 2.

**Table 2:** Immunosuppressive medications applied in autoimmunity and transplantation with their mechanisms, and impact on immune systems.

**Medications**	**Effect on immune cells, cytokines**	**Mechanism**	**Clinical applications**
Steroid (Prednisolone/ Budesonide)	Broad suppression of pro-inflammatory cytokines (74, 75)	Bind their cytosolic glucocorticoid receptor, translocate to the nucleus, and inhibit NF-κB-mediated transcription	Organ transplants Autoimmune diseases
Mycophenolate mofetil (MMF)	Purine is required for proliferation of T cells and B cells	Block enzyme IMPDH resulting in inhibition of *de novo* purine synthesis (76)Down-regulate co-stimulatory molecules on dendritic cells (77)	Organ transplants Autoimmune diseases
Calcineurin inhibitors (Tacrolimus/FK506) Cyclosporine A	IL-2 is crucial cytokines for Treg and T effectors survival and function (25)	Inhibit intracellular phosphatase calcineurin thus impair IL-2 production (78, 79)	Organ transplants
JAK3 inhibitor (Tofacitinib)	JAK3 signaling is critical to normal homeostasis and function of T cells, B cells, and NK cells; SCID in JAK3 mutations	JAK3 transduces signals downstream of CD132, which is the common gamma chain	Autoimmune diseases (80–83)
mTOR inhibitors Sirolimus (more effective for mTORC1), Everolimus	T cells differentiation (84, 85) Selective Treg expansion compared to T conventional cell in cell culture (86)	Inhibit downstream of PI3K and Akt via mTORC1 and mTORC2 mTORC1 is required for Treg activation (87)	Organ transplants
Anti-TNF Infliximab	Promote activation of innate and adaptive immunity	Pro-inflammatory cytokine	Rheumatoid arthritis Autoimmune hepatitis
Anti-IL6 R Tocilizumab	IL-6 leads to conventional T cells resistant to Treg suppression, Destabilizes Treg by inhibiting Foxp3 expression (88)	Pro-inflammatory cytokine promotes B and T cell proliferation and differentiation	Rheumatoid arthritis
Chemokine Receptor e.g. CXCR3	Both Treg and T effector cells express CXCR3 (58, 89)	Migrate to inflamed tissue where expression of CXCL9, (9, 10)	Autoimmune diseases
Anti-CD25 Basiliximab Daclizumab	Suppress immune response by targeting recently activated effector T cells that express CD25	Block the IL-2-binding site of CD25 (90)	Organ transplants
Rituximab	Depletes B cells	Anti-CD20 mAb	Antibody-mediated rejection, ABO-incompatible kidney transplants (91) Autoimmune hepatitis (92) GVHD (93) SLE

*Inosine monophosphate dehydrogenase (IMPDH), phosphatidylinositol 3-kinase (PI3K), Akt. mTOR complex 1 (mTORC1) and mTOR complex 2 (mTORC2), Janus associated kinase 3 (JAK3)*.

If transplant tolerance is to be achieved, it is essential that early inflammatory responses to allograft tissue must be regulated (94). In comparison to other organ transplantations, the liver is uniquely tolerant, which was first evidenced in 1969 by Sir Roy Calne, who demonstrated that liver allografts were accepted across MHC mismatch in pigs without immunosuppression (95). CD4^+^CD25^+^Foxp3^+^ Treg cells function by maintaining immunological self-tolerance of the allograft, immune homeostasis, and suppression of immune responses in order to reduce the destruction of tissue and support the allograft (96). Within a transplanted liver and hepatic draining lymph nodes, there is highly complex immune cells interplay between effector T cells, Treg cells, and antigen presenting dendritic cells (69). Dysregulation of effector T cell response to transplanted antigens and failure of Treg cells to suppress donor antigen-specific T effector cells leads to transplant rejection.

Todo and colleagues first described the application of Treg cells in liver transplantation. They infused autologous Treg-enriched cell populations (Treg cell frequency range from 2.6–16.9% of cell population) to ten living donor liver transplant recipients 2 weeks following transplantation. Treg cells from recipient lymphocytes are amplified by co-culture with irradiated donor cells in the presence of anti-CD80/CD86 monoclonal antibodies *in vitro* for 2 weeks. Additionally, liver recipients underwent splenectomy. Liver transplant patients received MMF, cyclosporine and tacrolimus during the post-transplant period. The study showed that seven out of ten patients were successfully withdrawn from immunosuppression, ranging from 6 to 18 months post-transplantation (97).

Furthermore, in a recent phase I clinical trial using CD4^+^CD25^+^Foxp3^+^ GMP-Treg cells, it was revealed that Treg cell administration was safe in liver transplantation. Peripheral blood Treg cells were isolated by leukapheresis and expanded under GMP conditions with IL-2 and rapamycin and subsequently administered to post-liver transplant patients at either 0.5–1 million/Kg or 3–4.5 million/kg. A total of nine patients (three patients less than 6 months after transplantation and six patients more than 6 months post-transplantation) received the GMP-Treg cells. The study showed that Treg cell therapy was safe and increased the pool of circulating Treg cells (98).

In addition to GMP-Treg cellular therapy, IL-2 has also been applied in liver transplant recipients to enhance Treg cell frequency and function. Treg cells constitutively express the high affinity IL-2 receptor, which makes them exquisitely sensitive to very low-doses of IL-2. A clinical trial to test the capacity of low-dose IL-2 in order to promote the selective expansion of endogenous Treg cells in liver transplant recipients is still on-going and results are awaited (LITE trial, NCT02949492).

## Immunosuppressive Medications and Regulatory T Cells

Either single or combination of immunosuppressive medications is normally applied in both AIDs and following organ transplantations. Calcineurin inhibitors (Tacrolimus and cyclosporine), mycophenolate mofetil (MMF or myfortif), Sirolimus, and corticosteroids are commonly used immunosuppressive drugs in both contexts. Rituximab, B cells depleting therapy, is also widely used in SLE and recently in difficult-to-treat AIH. Anti-cytokine therapy (anti-TNF and anti-IL-6) and JAK inhibitors have been applied in AIDs. Table 2 describes the types of immunosuppressive medications, their impact on immune cells and their mechanisms of action (99).

## Development Over Last Decade

### Target Tissue Homing and Tracking of Tregs

In order for Treg cells to exert their suppressive ability to tissue damaging effector T cells, cellular migration to the target tissue is essential. A recent study by our laboratory demonstrated that 22–44% of indium labeled GMP clinical grade Treg cells could be detected in the inflamed human liver by SPECT CT scan (60). These GMP Treg cells had high levels of CXCR3 expression. CXCR3 ligands (CXCL9, CXCL10, CXCL11) are expressed on the sinusoids, hepatocytes, and bile ducts in the inflamed liver (60, 100). Thus, up-regulating chemokine receptors on the Treg cells or enhancing chemokine expression at the target tissue site would be an option to achieve homing of Treg cells in both organ specific AID and transplantation. Labeling of GMP Treg cells with deuterium can track the infused cells in the peripheral circulation (38) and hence can be applied to track cells for longitudinal phenotyping. Labeling of GMP Treg cells with indium and tracking the cells in real time with SPECT-CT scan would be an alternative way to reassure that cells that express tissue specific chemokine receptors migrate to the target tissue (60).

Current Treg cell manufacturing technologies are only available for large-scale polyclonal Treg cell production. Polyclonal Treg cells can be expanded using anti-CD3 and anti-CD28-coated beads and subsequent supplementation with IL-2 (101). The use of large numbers of polyclonal Treg cells with unknown antigen specificities has led to unwanted effects, such as systemic immunosuppression, which can be avoided via utilization of antigen-specific Treg cells.

### Antigen Specificity and Chimeric Antigenic Receptor Tregs

Antigen-specific chimeric antigen receptor (CAR)-Treg cells have an advantage compared to polyclonal Treg cells due to their ability to migrate to the target organ, which express a specific antigen. Antigen-specific Treg cells have been shown to be functionally superior to polyclonal Treg cells in animal models, for example, Tang and colleagues isolated and expanded Treg cells derived from a transgenic mice that expressed islet cell specific TCR (102). In addition, antigen-specific CAR-Treg cells migrate specifically to the site of the antigen, thus has the advantage of less global immunosuppression compared to polyclonal Treg cells. CAR Treg cells could be developed to redirect Tregs toward a specific donor leucocyte antigen (HLA) class I molecule (HLA-A2) that is expressed in grafts.

Autologous or allogenic choice of Treg cell will influence the timing of administration for cell therapy. Donor-derived or third-party cells, such as umbilical cord blood derived Treg cells, may be used to generate CAR-Treg cells rapidly in batch production, however they could be immunogenic. Yet, autologous CAR-Treg cells, although not immunogenic, are produced individually, which is time-consuming (31). Other major challenges of Treg cell immunotherapy is the manufacturing of a large number of Treg cells to provide timely delivery of therapy, the efficacious dose of cells, the frequency of administration and recently, a major question being, what role does the low dose of IL-2 play in enhancing survival and function of polyclonal and antigen-specific Treg cells (28).

There are different approaches to obtain antigen-specific Treg cells and these include conferring antigen expression via CARs or engineering Treg cells with TCRs, specificity via transfection of viral vectors encoding specific TCRs. Both types of cells are promising given that they preferentially migrate to target sites and exert more potent and specific immunosuppression than polyclonal Treg cells. Engineering Treg cells with TCRs is a promising approach but limitations occur due to MHC-restrictions. Alloantigen-reactive Treg cells can be expanded through donor APCs, such as dendritic cells, PBMCs, and B cells (103). Compared to Treg cells engineered with TCRs (TCR-Treg), CAR-modified Treg (CAR-Treg) cells engineered in a non-MHC restricted manner have the advantage of widespread application. Additionally, CAR-Treg cells are less dependent on IL-2 than TCR-Treg cells.

### Large Scale Treg Cell Production

**Figure 4:**
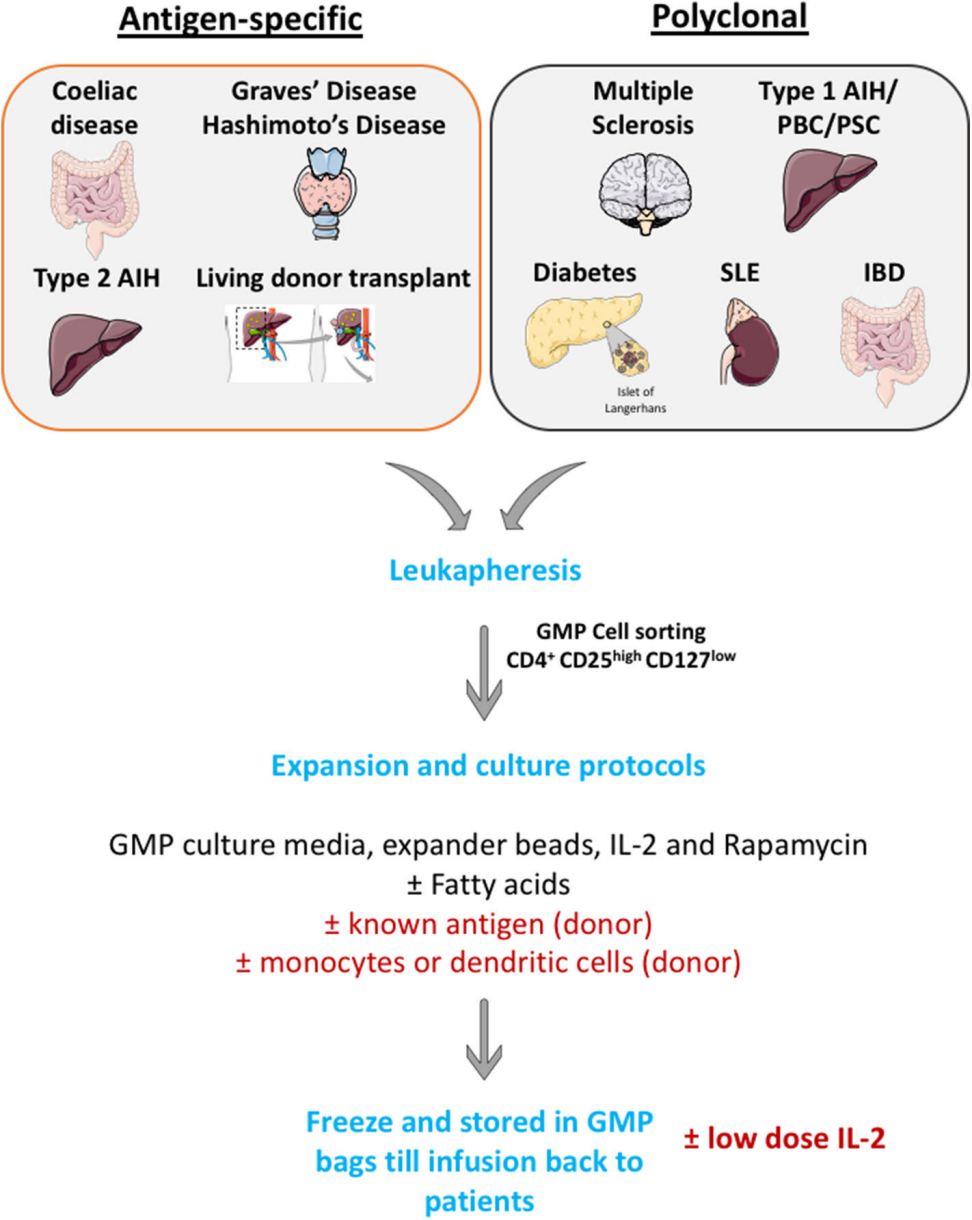
Schematic illustration of large scale GMP Treg production. Leukapharesis procedures are performed to those patients with either autoimmune diseases or those who will undergo transplantations. GMP Treg will be cell sorted using surface markers to obtain highly pure CD4^+^ CD25^high^ CD127^low^ cells population. Then, cells will be expanded in GMP culture media with GMP-grade IL-2 cytokines, GMP-grade rapamycin (to prevent effector T cells outgrowth) and GMP-grade CD3, CD28 Treg expander beads (with or without known antigen). These cells will then undergo quality assessment process and will be frozen until infusion back to patients.

Many investigators now have a GMP cell production facility and apply large scale Treg cell isolation and expansion. Cells are then frozen in GMP condition and thaw before infusing back to patients (Figure 4).

The quality of Treg cell function can be assessed before infusion by phenotyping isolated Treg cells to make sure that the purity is more than 95% (CD4^+^ CD25^high^ and CD127^low^), with a high level of transcription factor Foxp3 expression. Flow cytometry analysis can also be applied to assess functional markers of Treg (CD39, CTLA-4, IL-10) on the isolated and expanded Treg cells. In addition, quality of Treg cells can be assessed by epigenetic testing of isolated and expanded GMP Treg cells with analysis of Treg specific demethylated region (TSDR) with epigenetic analysis.

Treg therapeutic effect could be predicted by clinical readouts. For example, in the context of liver disease to assess whether Treg cell infusion could improve liver function tests (liver enzymes—alanine transaminase and aspartate transaminase, bilirubin) and in the context of diabetes (insulin requirement and c-peptide level improvement). Also, Treg therapeutic effect can be monitored with immunological readouts (e.g., decline in effector Th1 or CD8+ T cell frequency in the blood and assessing the frequency of cells in the tissue before and after infusion of cells with liver biopsy).

It is crucial to monitor the persistence of Treg cells when deciding the timing and frequency of Treg cells infusion. Treg cells can be monitored in both peripheral circulation and at tissue level. A recent study by our group showed Indium tropolonate labeling (60) and Deuterium labeling (38) can be applied to investigate the persistence of Treg cells in the circulation. Furthermore, persistence of infused Treg cells can sometimes be assessed in the tissue by performing a biopsy if it is ethically feasible and approved by the local team.

Treg cell function after infusion can be assessed directly by tracing the infused GMP Treg cells and conducting suppressive assays with autologous effector T cells, or indirectly by longitudinal phenotyping of effector T cells to investigate the decline in the frequency of these cells.

## Regulatory T Cells in Clinical Trials in Liver Transplantation and Autoimmune Diseases

Current Treg cell therapy use autologous cells. The drawback of using autologous cells is the time-delay to administer Treg cells from taking peripheral blood to obtaining sufficient numbers of cells. To achieve the Treg cell infusion at the time of transplantation, allogeneic Treg cells produced by umbilical cord blood would be a potential option. In addition, live donor transplantations (liver and kidney) would also allow the investigator to obtain sufficient numbers of functional Treg cells before the day of organ transplantation. It is crucial to obtain tissue biopsies following Treg infusion to access the localization of infused cells. A recent study focusing on skin biopsies of lupus lesions following Treg cell infusion suggested that there was a shift in Th1 to Th17 gene signature (44). In addition, the choice of immunosuppression in patients with Treg cell therapy is crucial, for example, rapamycin has been shown to enhance Treg cell frequency (104). To achieve efficacious and successful Treg therapy, it is necessary to continue on immunosuppression that is favorable to Treg cell survival and proliferation.

Application of CAR-Treg cells is an exciting option, in both transplantation and AID, where there is a known antigen. However, there are still major hurdles that must be overcome before CAR-Treg cells can be used in clinic. Antibodies specific for self- or alloantigen must be characterized to construct antigen-specific CAR-Treg cells. Additionally, it is crucial to achieve homing of CAR-Treg cells to the exact target site. Treatments with anti-tumor CAR-T cells cause side effects such as a cytokine “storm” and neuronal cytotoxicity (105). Yet, exhaustion of CAR Treg cells may limit their efficacy in immunosuppression. Therefore, more work is required to administer CAR-Treg cells effectively and safely to restore tolerance in transplantations and AIDs.

Plasticity of polyclonal or CAR-Treg cells in an inflamed microenvironment is still an unknown factor and concern to most investigators. Some data suggested that the plasticity and instability of cells in inflamed tissue and Treg cells could convert into pathogenic effector T cells (106, 107). The inflamed microenvironment enriched with pro-inflammatory cytokines can either lead to a reduction in the potency of Treg cells or resistance of T effector cells to Treg cell suppression (25). There are also questions to be addressed regarding the long-term proliferative potential and survival of polyclonal or antigen-specific Treg cells in the tissue microenvironment, which is enriched with cytokines, metabolites, low oxygen levels, and microbial peptides (in the context of liver) (107, 108). Additionally, metabolites and microbes from the portal vein toward the liver can have an impact on metabolism, phenotype, and function of intrahepatic Treg cells. These microenvironmental factors will determine the biology of GMP Treg cells at the specific tissue locations. Treg cells co-localize not only with effector CD4^+^ and CD8^+^ T cells but also with other immune cells, including antigen presenting dendritic cells, microbes-primed MAIT cells, phagocytic macrophages, innate NK cells, and neutrophils (109). The cross talk of Treg cells with other immune cells and microenvironmental factors will shape the phenotype and function of Treg cells; thus it is worth considering these factors for each tissue when designing Treg cell clinical trials (Figure 3).

## Extracellular Vesicles

Recently, it has been reported that intracellular communication of Treg cells can occur through extracellular vesicles (EVs), which include exosomes, apoptotic vesicles, and macrovesicles (110) Importantly, they appear to be involved in alloimmunity, thus, EVs may be an alternative therapeutic approach. EVs were identified in CD4^+^CD25^+^ Treg cells isolated from rodents, which were found to be produced after TCR activation. Smyth and colleagues identified that CD73 was present on Treg-derived EVs, which was essential for Treg cell mediated suppression. To support this, Yu and colleagues reported that in an *in vitro* kidney transplantation mouse model, Treg-derived EVs effectively suppressed T cell proliferation in a dose-dependent manner (111). Patients with relapsing-remitting multiple sclerosis had Treg-derived exosomes with impaired suppressive function (112, 113). It has been previously shown that in an autoimmune colitis model, Treg exosomes suppressed inflammatory T cell immunity, thereby preventing systemic inflammation, through transfer of IFN-y suppressing miRNA to T helper cells (114). Tung and colleagues identified that human Treg-derived EVs may be immune regulators and could be a potential treatment of transplant rejection (115). They also reported that TCR-activation of human Treg cells resulted in a release of EVs that efficiently suppressed T cell proliferation. Additionally, they identified that EVs had the ability to alter their cytokine profile to favor IL-10 and IL-4 secretion, whilst reducing expression of IL-6, IL-2, and IFN-y simultaneously (116). In line with this evidence, the group demonstrated that in a humanized mouse skin transplant model, human Treg-derived EVs were capable of protecting human skin grafts from alloimmune-mediated damage, through reducing immune cell infiltrate, thus supporting that EVs may be immune regulators, and thus an important therapeutic alternative. It is noteworthy that EVs derived from dendritic cells have previously been utilized in phase 2 clinical trials for non-small cell lung cancer therapy and were identified to be efficacious (116). This suggests that EVs may be a promising therapeutic option, however there are many limitations that would be necessary to address prior to EVs being utilized in a clinical setting, including, but not limiting to, the dose regimen, optimization of *ex vivo* isolation of GMP-grade Treg-derived EV protocols, and identification of *in vivo* targets.

## Future Direction of Regulatory T Cells in Transplantation and Autoimmunity

Treg cells have proved to be a major breakthrough as an exciting immunotherapy option in the last two decades. Early phase clinical trials demonstrated safety, feasibility, and early efficacy with GMP Tregs therapy in both autoimmune diseases and organ transplantation. Achieving successful Treg immunotherapy would lead to an immunosuppression-free period for patients. Development of antigen-specific Treg cells and CAR-Treg cells would lead to exciting new frontiers in the cell therapy field as these cells are more efficacious and lesser numbers of cells will be required due to their target tissue homing affinity. Over the last decade, manufacturing processes and culture media has been optimized. In addition, cytokines, such as IL-2, can support infused Treg cell survival and they would play as an adjuvant therapy in GMP Treg trials. Additionally, enhanced understanding of the patient's OMICs profile with new technologies will also allow us to apply personalized Treg cell immunotherapy. Although there are challenges, the future is exciting for the cell therapy community to collaborate closely in order to achieve a potentially effective immunotherapy and replace immunosuppression in both autoimmune disease and transplantation.

## Author Contributions

LT and YO conceptualize, wrote, edit, and approve the manuscript. All authors contributed to the article and approved the submitted version.

## Conflict of Interest

The authors declare that the research was conducted in the absence of any commercial or financial relationships that could be construed as a potential conflict of interest.

## References

[1] Sakaguchi S Sakaguchi N Asano M Itoh M Toda M. Immunologic self-tolerance maintained by activated T cells expressing IL-2 receptor alpha-chains (CD25). Breakdown of a single mechanism of self-tolerance causes various autoimmune diseases. J Immunol. (1995) 155:1151–64. 7636184

[2] Liu W Putnam A Xu-Yu Z Szot G Lee M Zhu S. CD127 expression inversely correlates with FoxP3 and suppressive function of human CD4+ T reg cells. J Exp Med. (2006) 203:1701–11. 10.1084/jem.20060772 16818678 PMC2118339

[3] Itoh M Takahashi T Sakaguchi N Kuniyasu Y Shimizu J Otsuka F. Thymus and autoimmunity: production of CD25+CD4+ naturally anergic and suppressive T cells as a key function of the thymus in maintaining immunologic self-tolerance. J Immunol. (1999) 162:5317–26. 10228007

[4] Hori S Nomura T Sakaguchi S. Control of regulatory T cell development by the transcription factor Foxp3. Science. (2003) 299:1057–61. 10.1126/science.1079490 12522256

[5] Vandenbark AA Offner H. Critical evaluation of regulatory T cells in autoimmunity: are the most potent regulatory specificities being ignored? Immunology. (2008) 125:1–13. 10.1111/j.1365-2567.2008.02900.x 18798915 PMC2526254

[6] Lyon M Peters J Glenister P Ball S Wright E. The scurfy mouse mutant has previously unrecognized hematological abnormalities and resembles Wiskott-Aldrich syndrome. Proc Natl Acad Sci USA. (1990) 87:2433–7. 10.1073/pnas.87.7.2433 2320565 PMC53703

[7] Seddiki N Santner-Nanan B Martinson J Zaunders J Sasson S Landay A. Expression of interleukin (IL)-2 and IL-7 receptors discriminates between human regulatory and activated T cells. J.Exp.Med. (2006) 203:1693–700. 10.1084/jem.20060468 16818676 PMC2118333

[8] Sakaguchi S Yamaguchi T Nomura T Ono M. Regulatory T cells and immune tolerance. Cell. (2008) 133:775–87. 10.1016/j.cell.2008.05.009 18510923

[9] Qureshi O Zheng Y Nakamura K Attridge K Manzotti C Schmidt E. Trans-endocytosis of CD80 and CD86: a molecular basis for the cell-extrinsic function of CTLA-4. Science. (2011) 332:600–3. 10.1126/science.1202947 21474713 PMC3198051

[10] Gratz I Truong H Yang S Maurano M Lee K Abbas AK. Cutting edge: memory regulatory t cells require IL-7 and not IL-2 for their maintenance in peripheral tissues. J Immunol. (2013) 190:4483–7. 10.4049/jimmunol.1300212 23543753 PMC3660612

[11] Abbas A Benoist C Bluestone J Campbell D Ghosh S Hori S. Regulatory T cells: recommendations to simplify the nomenclature. Nat Immunol. (2013) 14:307–8. 10.1038/ni.2554 23507634

[12] Polansky J Kretschmer K Freyer J Floess S Garbe A Baron U. DNA methylation controls Foxp3 gene expression. Eur J Immunol. (2008) 38:1654–63. 10.1002/eji.200838105 18493985

[13] Curotto de Lafaille MA Lafaille JJ Natural and adaptive foxp3+ regulatory T cells: more of the same or a division of labor? Immunity. (2009) 30:626–35. 10.1016/j.immuni.2009.05.002 19464985

[14] Kim K Hong S Han D Yi J Jung J Yang B. Dietary antigens limit mucosal immunity by inducing regulatory T cells in the small intestine. Science. (2016) 351:858–63. 10.1126/science.aac5560 26822607

[15] Steinman L Merrill J McInnes I Peakman M. Optimization of current and future therapy for autoimmune diseases. Nat Med. (2012) 18:59–65. 10.1038/nm.2625 22227674

[16] Chen W Jin W Hardegen N Lei K Li L Marinos N. Conversion of peripheral CD4+CD25- naive T cells to CD4+CD25+ regulatory T cells by TGF-beta induction of transcription factor Foxp3. J Exp Med. (2003) 198:1875–86. 10.1084/jem.20030152 14676299 PMC2194145

[17] Kretschmer K Apostolou I Hawiger D Khazaie K Nussenzweig M von Boehmer H. Inducing and expanding regulatory T cell populations by foreign antigen. Nat Immunol. (2005) 6:1219–27. 10.1038/ni1265 16244650

[18] Wing K Sakaguchi S. Regulatory T cells exert checks and balances on self tolerance and autoimmunity. Nat Immunol. (2010) 11:7–13. 10.1038/ni.1818 20016504

[19] Hartemann A Bensimon G Payan C Jacqueminet S Bourron O Nicolas N. Low-dose interleukin 2 in patients with type 1 diabetes: a phase 1/2 randomised, double-blind, placebo-controlled trial. Lancet Diabetes Endocrinol. (2013) 1:295–305. 10.1016/S2213-8587(13)70113-X 24622415

[20] Yang J Sherry R Steinberg S Topalian S Schwartzentruber D Hwu P. Randomized study of high-dose and low-dose interleukin-2 in patients with metastatic renal cancer. J Clin Oncol. (2003) 21:3127–32. 10.1200/JCO.2003.02.122 12915604 PMC2275327

[21] Klatzmann D Abbas AK. The promise of low-dose interleukin-2 therapy for autoimmune and inflammatory diseases. Nat Rev Immunol. (2015) 15:283–94. 10.1038/nri3823 25882245

[22] Taniguchi T Miyazaki T Minami Y Kawahara A Fujii H Nakagawa Y. IL-2 signaling involves recruitment and activation of multiple protein tyrosine kinases by the IL-2 receptor. Ann N Y Acad Sci. (1995) 766:235–44. 10.1111/j.1749-6632.1995.tb26671.x 7486666

[23] Hofer T Krichevsky O Altan-Bonnet G. Competition for IL-2 between regulatory and effector T cells to Chisel immune responses. Front Immunol. (2012) 3:268. 10.3389/fimmu.2012.00268 22973270 PMC3433682

[24] Busse D de la Rosa M Hobiger K Thurley K Flossdorf M Scheffold A. Competing feedback loops shape IL-2 signaling between helper and regulatory T lymphocytes in cellular microenvironments. Proc Natl Acad Sci USA. (2010) 107:3058–63. 10.1073/pnas.0812851107 20133667 PMC2840293

[25] Chen Y Jeffery H Hunter S Bhogal R Birtwistle J Braitch M. Human intrahepatic regulatory T cells are functional, require IL-2 from effector cells for survival, and are susceptible to Fas ligand-mediated apoptosis. Hepatology. (2016) 64:138–50. 10.1002/hep.28517 26928938 PMC4950043

[26] Jeffery H Jeffery L Lutz P Corrigan M Webb G Hirschfield G. Low-dose interleukin-2 promotes STAT-5 phosphorylation, Treg survival and CTLA-4-dependent function in autoimmune liver diseases. Clin Exp Immunol. (2017) 188:394–411. 10.1111/cei.12940 28176332 PMC5422719

[27] Oo YH Hubscher SG Adams DH. Autoimmune hepatitis: new paradigms in the pathogenesis, diagnosis, and management. Hepatol Int. (2010) 4:475–93. 10.1007/s12072-010-9183-5 20827405 PMC2900560

[28] Than NN Jeffery HC Oo YH Autoimmune hepatitis: progress from global immunosuppression to personalised regulatory T cell therapy. Can J Gastroenterol Hepatol. (2016) 2016:7181685 10.1155/2016/7181685 27446862 PMC4904688

[29] Oo YH Neuberger J. Options for treatment of primary biliary cirrhosis. Drugs. (2004) 64:2261–71. 10.2165/00003495-200464200-00001 15456326

[30] Miyara M Sakaguchi S. Human FoxP3(+)CD4(+) regulatory T cells: their knowns and unknowns. Immunol Cell Biol. (2011) 89:346–51. 10.1038/icb.2010.137 21301480

[31] Oo YH Sakaguchi S. Regulatory T-cell directed therapies in liver diseases. J Hepatol. (2013) 59:1127–34. 10.1016/j.jhep.2013.05.034 23727305

[32] Jeffery H Braitch M Brown S Oo Y. Clinical potential of regulatory T cell therapy in liver siseases: an overview and current perspectives. Front Immunol. (2016) 7:334. 10.3389/fimmu.2016.00334 27656181 PMC5012133

[33] Ronca V Mancuso C Milani C Carbone M Oo Y Invernizzi P. Immune system and cholangiocytes: a puzzling affair in primary biliary cholangitis. J Leukoc Biol. (2020) 108:659–71. 10.1002/JLB.5MR0320-200R 32349179

[34] Atif M Warner S Oo YH. Linking the gut and liver: crosstalk between regulatory T cells and mucosa-associated invariant T cells. Hepatol Int. (2018) 12:305–14. 10.1007/s12072-018-9882-x 30027532 PMC6097019

[35] Herman A Freeman G Mathis D Benoist C. CD4+CD25+ T regulatory cells dependent on ICOS promote regulation of effector cells in the prediabetic lesion. J Exp Med. (2004) 199:1479–89. 10.1084/jem.20040179 15184501 PMC2211778

[36] Walker LS Abbas AK. The enemy within: keeping self-reactive T cells at bay in the periphery. Nat Rev Immunol. (2002) 2:11–9. 10.1038/nri701 11908514

[37] Miyara M Gorochov G Ehrenstein M Musset L Sakaguchi S Amoura Z Human FoxP3+ regulatory T cells in systemic autoimmune diseases. Autoimmun Rev. (2011) 10:744–55. 10.1016/j.autrev.2011.05.004 21621000

[38] Bluestone J Buckner J Fitch M Gitelman S Gupta S Hellerstein M. Type 1 diabetes immunotherapy using polyclonal regulatory T cells. Sci Transl Med. (2015) 7:315ra189. 10.1126/scitranslmed.aad4134 26606968 PMC4729454

[39] Marek-Trzonkowska N Mysliwiec M Dobyszuk A Grabowska M Techmanska I Juscinska J. Administration of CD4+CD25highCD127- regulatory T cells preserves beta-cell function in type 1 diabetes in children. Diabetes Care. (2012) 35:1817–20. 10.2337/dc12-0038 22723342 PMC3425004

[40] MacDonald K Hoeppli R Huang Q Gillies J Luciani D Orban P. Alloantigen-specific regulatory T cells generated with a chimeric antigen receptor. J Clin Invest. (2016) 126:1413–24. 10.1172/JCI82771 26999600 PMC4811124

[41] Boardman D Philippeos C Fruhwirth G Ibrahim M Hannen R Cooper D. Expression of a chimeric antigen receptor specific for donor HLA class I enhances the potency of human regulatory T cells in preventing human skin transplant rejection. Am J Transplant. (2017) 17:931–43. 10.1111/ajt.14185 28027623

[42] Noyan F Zimmermann K Hardtke-Wolenski M Knoefel A Schulde E Geffers R. Prevention of allograft rejection by use of regulatory T cells with an MHC-specific Chimeric antigen receptor. Am J Transplant. (2017) 17:917–30. 10.1111/ajt.14175 27997080

[43] Esensten J Muller Y Bluestone J Tang Q. Regulatory T-cell therapy for autoimmune and autoinflammatory diseases: the next frontier. J Allergy Clin Immunol. (2018) 142:1710–18. 10.1016/j.jaci.2018.10.015 30367909

[44] Dall'Era M Pauli M Remedios K Taravati K Sandova P Putnam A. Adoptive treg cell therapy in a patient with systemic lupus erythematosus. Arthritis Rheumatol. (2019) 71:431–40. 10.1002/art.40737 30277008 PMC6447289

[45] Marek-Trzonkowska N Mysliwiec M Dobyszuk A Grabowska M Derkowska I Juścińska J. Therapy of type 1 diabetes with CD4(+)CD25(high)CD127-regulatory T cells prolongs survival of pancreatic islets—results of one year follow-up. Clin Immunol. (2014) 153:23–30. 10.1016/j.clim.2014.03.016 24704576

[46] Canavan J Scotta C Vossenkamper A Goldberg R Elder M Shoval I. Developing in vitro expanded CD45RA+ regulatory T cells as an adoptive cell therapy for Crohn's disease. Gut. (2016) 65:584–94. 10.1136/gutjnl-2014-306919 25715355 PMC4819603

[47] Saadoun D Rosenzwajg M Joly F Six A Carrat F Thibault V. Regulatory T-cell responses to low-dose interleukin-2 in HCV-induced vasculitis. N Engl J Med. (2011) 365:2067–77. 10.1056/NEJMoa1105143 22129253

[48] He J Zhang R Shao M Zhao X Miao M Chen J. Efficacy and safety of low-dose IL-2 in the treatment of systemic lupus erythematosus: a randomised, double-blind, placebo-controlled trial. Ann Rheum Dis. (2020) 79:141–9. 10.1136/annrheumdis-2019-215396 31537547 PMC6937406

[49] Rosenzwajg M Lorenzon R Cacoub P Pham H Pitoiset F Soufi K. Immunological and clinical effects of low-dose interleukin-2 across 11 autoimmune diseases in a single, open clinical trial. Ann Rheum Dis. (2019) 78:209–17. 10.1136/annrheumdis-2018-214229 30472651

[50] Todd J Evangelou M Cutler A Pekalski M Walker N Stevens H. Regulatory T cell responses in participants with Type 1 diabetes after a single dose of interleukin-2: a non-randomised, open label, adaptive dose-finding trial. PLoS Med. (2016) 13:e1002139. 10.1371/journal.pmed.1002139 27727279 PMC5058548

[51] Sebode M Peiseler M Franke B Schwinge D Schoknecht T Wortmann F. Reduced FOXP3(+) regulatory T cells in patients with primary sclerosing cholangitis are associated with IL2RA gene polymorphisms. J Hepatol. (2014) 60:1010–6. 10.1016/j.jhep.2013.12.027 24412607

[52] Beuers U Gershwin M Gish R Invernizzi P Jones D Lindor K. Changing nomenclature for PBC: From 'cirrhosis' to 'cholangitis'. Hepatology. (2015) 62:1620–2. 10.1016/j.clinre.2015.08.001 26372460

[53] Oo YH Adams DH Regulatory T cells and autoimmune hepatitis: defective cells or a hostile environment? J Hepatol. (2012) 57:6–8. 10.1016/j.jhep.2012.04.005 22522382

[54] Oo YH Adams DH. Regulatory T cells and autoimmune hepatitis: what happens in the liver stays in the liver. J Hepatol. (2014) 61:973–5. 10.1016/j.jhep.2014.08.005 25131772

[55] Longhi M Hussain M Mitry R Arora S Mieli-Vergani G Vergani D. Functional study of CD4+CD25+ regulatory T cells in health and autoimmune hepatitis. J.Immunol. (2006) 176:4484–91. 10.4049/jimmunol.176.7.4484 16547287

[56] Oo YH Adams DH. The role of chemokines in the recruitment of lymphocytes to the liver. J Autoimmun. (2010) 34:45–54. 10.1016/j.jaut.2009.07.011 19744827

[57] Adams DH Lloyd AR. Chemokines: leucocyte recruitment and activation cytokines. Lancet. (1997) 349:490–5. 9040590 10.1016/s0140-6736(96)07524-1

[58] Oo Y Weston C Lalor P Curbishley S Withers D Reynolds G. Distinct roles for CCR4 and CXCR3 in the recruitment and positioning of regulatory T cells in the inflamed human liver. J Immunol. (2010) 184:2886–98. 10.4049/jimmunol.0901216 20164417

[59] Erhardt A Wegscheid C Claass B Carambia A Herkel J Mittrücker H. CXCR3 deficiency exacerbates liver disease and abrogates tolerance in a mouse model of immune-mediated hepatitis. J Immunol. (2011) 186:5284–93. 10.4049/jimmunol.1003750 21441449

[60] Oo Y Ackrill S Cole R Jenkins L Anderson P Jeffery H. Liver homing of clinical grade Tregs after therapeutic infusion in patients with autoimmune hepatitis. JHEP Rep. (2019) 1:286–96. 10.1016/j.jhepr.2019.08.001 32039380 PMC7001578

[61] Lim T Martinez-Llordella M Kodela E Gray E Heneghan M Sanchez-Fueyo A. Low-dose interleukin-2 for refractory autoimmune hepatitis. Hepatology. (2018) 68:1649–52. 10.1002/hep.30059 29698571

[62] Battula N Platto M Anbarasan R Perera M Ong E Roll G. Intention to split policy: a successful strategy in a combined pediatric and adult liver transplant center. Ann Surg. (2017) 265:1009–15. 10.1097/SLA.0000000000001816 27257738

[63] Hornick P Lechler R. Direct and indirect pathways of alloantigen recognition: relevance to acute and chronic allograft rejection. Nephrol Dial Transplant. (1997) 12:1806–10. 9306324 10.1093/ndt/12.9.1806

[64] Brunstein C Miller J McKenna D Hippen K DeFor T Sumstad D. Umbilical cord blood-derived T regulatory cells to prevent GVHD: kinetics, toxicity profile, and clinical effect. Blood. (2016) 127:1044–51. 10.1182/blood-2015-06-653667 26563133 PMC4768428

[65] Brunstein C Miller J Cao Q McKenna D Hippen K Curtsinger J. Infusion of ex vivo expanded T regulatory cells in adults transplanted with umbilical cord blood: safety profile and detection kinetics. Blood. (2011) 117:1061–70. 10.1182/blood-2010-07-293795 20952687 PMC3035067

[66] Edinger M Hoffmann P Ermann J Drago K Fathman C Strober S. CD4+CD25+ regulatory T cells preserve graft-versus-tumor activity while inhibiting graft-versus-host disease after bone marrow transplantation. Nat Med. (2003) 9:1144–50. 10.1038/nm915 12925844

[67] Martelli M Di Ianni M Ruggeri L Falzetti F Carotti A Terenzi A. HLA-haploidentical transplantation with regulatory and conventional T-cell adoptive immunotherapy prevents acute leukemia relapse. Blood. (2014) 124:638–44. 10.1182/blood-2014-03-564401 24923299

[68] Mathew J H-Voss J LeFever A Konieczna I Stratton C He J. A phase I clinical trial with ex vivo expanded recipient regulatory T cells in living donor kidney transplants. Sci Rep. (2018) 8:7428. 10.1038/s41598-018-25574-7 29743501 PMC5943280

[69] Chandran S Tang Q Sarwal M Laszik Z Putnam A Lee K. Polyclonal regulatory T cell therapy for control of inflammation in kidney transplants. Am J Transplant. (2017) 17:2945–54. 10.1111/ajt.14415 28675676 PMC5662482

[70] Feng S Ekong U Lobritto S Demetris A Roberts J Rosenthal P. Complete immunosuppression withdrawal and subsequent allograft function among pediatric recipients of parental living donor liver transplants. JAMA. (2012) 307:283–93. 10.1001/jama.2011.2014 22253395

[71] Ramos H Reyes J Abu-Elmagd K Zeevi A Reinsmoen N Tzakis A. Weaning of immunosuppression in long-term liver transplant recipients. Transplantation. (1995) 59:212–7. 7839442 10.1097/00007890-199501270-00010 PMC3005337

[72] Sagoo P Ali N Garg G Nestle F Lechler R Lombardi G. Human regulatory T cells with alloantigen specificity are more potent inhibitors of alloimmune skin graft damage than polyclonal regulatory T cells. Sci Transl Med. (2011) 3:83ra42. 10.1126/scitranslmed.3002076 21593402 PMC3776382

[73] Tang Q Bluestone JA. Regulatory T-cell therapy in transplantation: moving to the clinic. Cold Spring Harb Perspect Med. (2013) 3:a015552. 10.1101/cshperspect.a015552 24186492 PMC3808774

[74] Vandevyver S Dejager L Tuckermann J Libert C. New insights into the anti-inflammatory mechanisms of glucocorticoids: an emerging role for glucocorticoid-receptor-mediated transactivation. Endocrinology. (2013) 154:993–1007. 10.1210/en.2012-2045 23384835

[75] McKay LI Cidlowski JA. Molecular control of immune/inflammatory responses: interactions between nuclear factor-kappa B and steroid receptor-signaling pathways. Endocr Rev. (1999) 20:435–59. 10.1210/edrv.20.4.0375 10453354

[76] Silverman Kitchin J Pomeranz M Pak G Washenik K Shupack J. Rediscovering mycophenolic acid: a review of its mechanism, side effects, and potential uses. J Am Acad Dermatol. (1997) 37:445–9. 10.1016/s0190-9622(97)70147-6 9308561

[77] Mehling A Grabbe S Voskort M Schwarz T Luger T Beissert S. Mycophenolate mofetil impairs the maturation and function of murine dendritic cells. J Immunol. (2000) 165:2374–81. 10.4049/jimmunol.165.5.2374 10946260

[78] Moes A Hesselink D Zietse R van Schaik R van Gelder T Hoorn E. Calcineurin inhibitors and hypertension: a role for pharmacogenetics? Pharmacogenomics. (2014) 15:1243–51. 10.2217/pgs.14.87 25141899

[79] Giese T Zeier M Schemmer P Uhl W Schoels M Dengler T. Monitoring of NFAT-regulated gene expression in the peripheral blood of allograft recipients: a novel perspective toward individually optimized drug doses of cyclosporine A. Transplantation. (2004) 77:339–44. 10.1097/01.TP.0000109260.00094.01 14966405

[80] Xing L Dai Z Jabbari A Cerise J Higgins C Gong W. Alopecia areata is driven by cytotoxic T lymphocytes and is reversed by JAK inhibition. Nat Med. (2014) 20:1043–9. 10.1038/nm.3645 25129481 PMC4362521

[81] Tavakolpour S. Tofacitinib as the potent treatment for refractory pemphigus: a possible alternative treatment for pemphigus. Dermatol Ther. (2018) 31:e12696. 10.1111/dth.12696 30207045

[82] Kennedy Crispin M Ko J Craiglow B Li S Shankar G Urban J. Safety and efficacy of the JAK inhibitor tofacitinib citrate in patients with alopecia areata. JCI Insight. (2016) 1:e89776. 10.1172/jci.insight.89776 27699252 PMC5033755

[83] Hodge JA Kawabata TT Krishnaswami S Clark JD Telliez JB Dowty ME. The mechanism of action of tofacitinib—an oral Janus kinase inhibitor for the treatment of rheumatoid arthritis. Clin Exp Rheumatol. (2016) 34:318–28. 26966791

[84] Delgoffe G Pollizzi K Waickman A Heikamp E Meyers D Horton M. The kinase mTOR regulates the differentiation of helper T cells through the selective activation of signaling by mTORC1 and mTORC2. Nat Immunol. (2011) 12:295–303. 10.1038/ni.2005 21358638 PMC3077821

[85] Araki K Turner A Shaffer V Gangappa S Keller S Bachmann M. mTOR regulates memory CD8 T-cell differentiation. Nature. (2009) 460:108–12. 10.1038/nature08155 19543266 PMC2710807

[86] Hippen K Merkel S Schirm D Sieben C Sumstad D Kadidlo D. Massive ex vivo expansion of human natural regulatory T cells (T(regs)) with minimal loss of in vivo functional activity. Sci Transl Med. (2011) 3:83ra41. 10.1126/scitranslmed.3001809 21593401 PMC3551476

[87] Zeng H Yang K Cloer C Neale G Vogel P Chi H. mTORC1 couples immune signals and metabolic programming to establish T(reg)-cell function. Nature. (2013) 499:485–90. 10.1038/nature12297 23812589 PMC3759242

[88] Samanta A Li B Song X Bembas K Zhang G Katsumata M. TGF-beta and IL-6 signals modulate chromatin binding and promoter occupancy by acetylated FOXP3. Proc Natl Acad Sci USA. (2008) 105:14023–7. 10.1073/pnas.0806726105 18779564 PMC2544572

[89] Oo Y Banz V Kavanagh D Liaskou E Withers D Humphreys E. CXCR3-dependent recruitment and CCR6-mediated positioning of Th-17 cells in the inflamed liver. J Hepatol. (2012) 57:1044–51. 10.1016/j.jhep.2012.07.008 22796894 PMC3994510

[90] Pascual J Marcen R Ortuno J. Anti-interleukin-2 receptor antibodies: basiliximab and daclizumab. Nephrol Dial Transplant. (2001) 16:1756–60. 10.1093/ndt/16.9.1756 11522853

[91] Clatworthy MR. Targeting B cells and antibody in transplantation. Am J Transplant. (2011) 11:1359–67. 10.1111/j.1600-6143.2011.03554.x 21668625 PMC4148618

[92] Than N Hodson J Schmidt-Martin D Taubert R Wawman R Botter M. Efficacy of rituximab in difficult-to-manage autoimmune hepatitis: results from the International Autoimmune Hepatitis Group. JHEP Rep. (2019) 1:437–45. 10.1016/j.jhepr.2019.10.005 32039395 PMC7005655

[93] Kim S Lee J Jung C Min C Cho B Shin H. Weekly rituximab followed by monthly rituximab treatment for steroid-refractory chronic graft-versus-host disease: results from a prospective, multicenter, phase II study. Haematologica. (2010) 95:1935–42. 10.3324/haematol.2010.026104 20663943 PMC2966917

[94] Salisbury EM Game DS Lechler RI. Transplantation tolerance. Pediatr Nephrol. (2014) 29:2263–72. 10.1007/s00467-013-2659-5 24213880 PMC4212135

[95] Calne R Sells R Pena J Davis D Millard P Herbertson B. Induction of immunological tolerance by porcine liver allografts. Nature. (1969) 223:472–6. 10.1038/223472a0 4894426

[96] Tang Q Bluestone JA. The Foxp3+ regulatory T cell: a jack of all trades, master of regulation. Nat Immunol. (2008) 9:239–44. 10.1038/ni1572 18285775 PMC3075612

[97] Todo S Yamashita K Goto R Zaitsu M Nagatsu A Oura T. A pilot study of operational tolerance with a regulatory T-cell-based cell therapy in living donor liver transplantation. Hepatology. (2016) 64:632–43. 10.1002/hep.28459 26773713

[98] Sanchez-Fueyo A Whitehouse G Grageda N Cramp M Lim T Romano M. Applicability, safety, and biological activity of regulatory T cell therapy in liver transplantation. Am J Transplant. (2020) 20:1125–36. 10.1111/ajt.15700 31715056 PMC7154724

[99] Furukawa A Wisel SA Tang Q. Impact of immune-modulatory drugs on regulatory T cell. Transplantation. (2016) 100:2288–300. 10.1097/TP.0000000000001379 27490409 PMC5077666

[100] Curbishley S Eksteen B Gladue R Lalor P Adams D. CXCR 3 activation promotes lymphocyte transendothelial migration across human hepatic endothelium under fluid flow. Am J Pathol. (2005) 167:887–99. 10.1016/S0002-9440(10)62060-3 16127166 PMC1698725

[101] Putnam A Brusko T Lee M Liu W Szot G Ghosh T. Expansion of human regulatory T-cells from patients with type 1 diabetes. Diabetes. (2009) 58:652–62. 10.2337/db08-1168 19074986 PMC2646064

[102] Spence A Purtha W Tam J Dong S Kim Y Ju C. Revealing the specificity of regulatory T cells in murine autoimmune diabetes. Proc Natl Acad Sci USA. (2018) 115:5265–70. 10.1073/pnas.1715590115 29712852 PMC5960284

[103] Duggleby R Danby R Madrigal J Saudemont A. Clinical grade regulatory CD4(+) T cells (Tregs): moving toward cellular-based immunomodulatory therapies. Front Immunol. (2018) 9:252. 10.3389/fimmu.2018.00252 29487602 PMC5816789

[104] Battaglia M Stabilini A Roncarolo MG. Rapamycin selectively expands CD4+CD25+FoxP3+ regulatory T cells. Blood. (2005) 105:4743–8. 10.1182/blood-2004-10-3932 15746082

[105] Cao J Wang H Gao W You J Wu L Wang Z. The incidence of cytokine release syndrome and neurotoxicity of CD19 chimeric antigen receptor-T cell therapy in the patient with acute lymphoblastic leukemia and lymphoma. Cytotherapy. (2020) 22:214–26. 10.1016/j.jcyt.2020.01.015 32305113

[106] Itoh S Kimura N Axtell R Velotta J Gong Y Wang X. Interleukin-17 accelerates allograft rejection by suppressing regulatory T cell expansion. Circulation. (2011) 124:S187–96. 10.1161/CIRCULATIONAHA.110.014852 21911812

[107] Jeffery H Hunter S Humphreys E Bhogal R Wawman R Birtwistle J. Bidirectional cross-talk between biliary epithelium and Th17 cells promotes local Th17 expansion and bile duct proliferation in biliary liver diseases. J Immunol. (2019) 203:1151–9. 10.4049/jimmunol.1800455 31391236 PMC6697739

[108] Wawman RE Bartlett H Oo YH. Regulatory T cell metabolism in the hepatic microenvironment. Front Immunol. (2017) 8:1889. 10.3389/fimmu.2017.01889 29358934 PMC5766647

[109] Jeffery H van Wilgenburg B Kurioka A Parekh K Stirling K Roberts S. Biliary epithelium and liver B cells exposed to bacteria activate intrahepatic MAIT cells through MR1. J Hepatol. (2016) 64:1118–27. 10.1016/j.jhep.2015.12.017 26743076 PMC4822535

[110] Samir EL Andaloussi Mäger I Breakefield XO Matthew JA Wood. Extracellular vesicles: biology and emerging therapeutic opportunities. Nat Rev Drug Discov. (2013) 12:347–57. 10.1038/nrd397 23584393

[111] Smyth L Ratnasothy K Tsang J Boardman D Warley A Lechler R. CD73 expression on extracellular vesicles derived from CD4+ CD25+ Foxp3+ T cells contributes to their regulatory function. Eur J Immunol. (2013) 43:2430–40. 10.1002/eji.201242909 23749427

[112] Yu X Huang C Song B Xiao Y Fang M Feng J. CD4+CD25+ regulatory T cells-derived exosomes prolonged kidney allograft survival in a rat model. Cell Immunol. (2013) 285:62–8. 10.1016/j.cellimm.2013.06.010 24095986

[113] Azimi M Ghabaee M Moghadasi AN Noorbakhsh F Izad M. Immunomodulatory function of Treg-derived exosomes is impaired in patients with relapsing-remitting multiple sclerosis. Immunol Res. (2018) 66:513–20. 10.1007/s12026-018-9008-5 29882035

[114] Okoye I Coomes S Pelly V Czieso S Papayannopoulos V Tolmachova T MicroRNA-containing T-regulatory-cell-derived exosomes suppress pathogenic T helper 1 cells. Immunity. (2014) 41:89–103. 10.1016/j.immuni.2014.05.019 25035954 PMC4104030

[115] Tung S Fanelli G Matthews R Bazoer J Letizia M Vizcay-Barrena G. Regulatory T cell extracellular vesicles modify t-effector cell cytokine production and protect against human skin allograft damage. Front Cell Dev Biol. (2020) 8:317. 10.3389/fcell.2020.00317 32509778 PMC7251034

[116] Besse B Charrier M Lapierre V Dansin E Lantz O Planchard D. Dendritic cell-derived exosomes as maintenance immunotherapy after first line chemotherapy in NSCLC. Oncoimmunology. (2015) 5:e1071008. 10.1080/2162402X.2015.1071008 27141373 PMC4839329

